# Characterizing the effect of expression of an acetyl-CoA synthetase insensitive to acetylation on co-utilization of glucose and acetate in batch and continuous cultures of *E. coli* W

**DOI:** 10.1186/s12934-018-0955-2

**Published:** 2018-07-09

**Authors:** Katharina Novak, Lukas Flöckner, Anna Maria Erian, Philipp Freitag, Christoph Herwig, Stefan Pflügl

**Affiliations:** 1 Research Area Biochemical Engineering, Institute for Chemical, Environmental and Bioscience Engineering, Technische Universität Wien, Gumpendorfer Straße 1a, 1060 Vienna, Austria; 2 Christian Doppler Laboratory for Mechanistic and Physiological Methods for Improved Bioprocesses, Gumpendorfer Straße 1a, 1060 Vienna, Austria

**Keywords:** *E. coli* W, Acetyl-CoA-synthetase, Acetate (Acs), Metabolic engineering, Protein acetylation, Biomass composition, Mixed feed system, Continuous cultures, Gene expression analysis, Acetate permease (ActP)

## Abstract

**Background:**

Due to its high stress tolerance and low acetate secretion, *Escherichia coli* W is reported to be a good production host for several metabolites and recombinant proteins. However, simultaneous co-utilization of glucose and other substrates such as acetate remains a challenge. The activity of acetyl-CoA-synthetase, one of the key enzymes involved in acetate assimilation is tightly regulated on a transcriptional and post-translational level. The aim of this study was to engineer *E. coli* W for overexpression of an acetylation insensitive acetyl-CoA-synthetase and to characterize this strain in batch and continuous cultures using glucose, acetate and during co-utilization of both substrates.

**Results:**

*Escherichia coli* W engineered to overexpress an acetylation-insensitive acetyl-CoA synthetase showed a 2.7-fold increase in acetate uptake in a batch process containing glucose and high concentrations of acetate compared to a control strain, indicating more efficient co-consumption of glucose and acetate. When acetate was used as the carbon source, batch duration could significantly be decreased in the overexpression strain, possibly due to alleviation of acetate toxicity. Chemostat cultivations with different dilution rates using glucose revealed only minor differences between the overexpression and control strain. Accelerostat cultivations using dilution rates between 0.20 and 0.70 h^−1^ indicated that *E. coli* W is naturally capable of efficiently co-utilizing glucose and acetate over a broad range of specific growth rates. Expression of acetyl-CoA synthetase resulted in acetate and glucose accumulation at lower dilution rates compared to the control strain. This observation can possibly be attributed to a higher ratio between *acs* and *pta*-*ackA* in the overexpression strain as revealed by gene expression analysis. This would result in enhanced energy dissipation caused by an imbalance in the Pta-AckA-Acs cycle. Furthermore, *yjcH* and *actP*, genes co-transcribed with acetyl-CoA synthetase showed significant down-regulation at elevated dilution rates.

**Conclusions:**

*Escherichia coli* W expressing an acetylation-insensitive acetyl-CoA synthetase was shown to be a promising candidate for mixed feed processes using glucose and acetate. Comparison between batch and continuous cultures revealed distinct differences in glucose-acetate co-utilization behavior, requiring additional investigations such as multi-omics analysis and further engineering towards even more efficient co-utilization strains of *E. coli* W.

**Electronic supplementary material:**

The online version of this article (10.1186/s12934-018-0955-2) contains supplementary material, which is available to authorized users.

## Background

*Escherichia coli* is among the best-studied organisms today and a workhorse of biotechnology used for the production of recombinant proteins [1–3] and fuel and bulk chemicals including ethanol [4, 5], isobutanol [6, 7] and 2,3-butanediol [8–10]. In particular, *E. coli* W has been described as good production host for industrial applications due to high stress tolerance [11, 12], fast growth up to high cell densities on various substrates including sucrose [13–15] and low acetate excretion [14].

Most studies utilize glucose as the carbon source, making glucose the best studied substrate for *E. coli* [12, 16]. However, other substrates such as pentoses [12, 17, 18], glycerol [19] and acetate [20, 21] have also been studied.

Acetate comprises an interesting alternative carbon source as it is a cheap industrial waste product contained in a broad variety of materials [22]. For instance, acetate is produced by anaerobic digestion of biomass from waste [23], during syngas fermentations [22, 24] and preparation of lignocellulosic hydrolysates [25]. Examples of acetate utilization for production of chemicals using *E. coli* W include succinic acid [26], itaconic acid [27] and isobutanol [28].

Co-utilization with glucose, a sugar abundantly available in a variety of potential substrate streams, would be an interesting option to increase competitiveness of an industrial process.

*Escherichia coli* produces acetate via different pathways, with the main route being the phosphate acetyltransferase (Pta) and acetate kinase (AckA) node. Others include direct oxidation of pyruvate to acetate and CO_2_ by pyruvate dehydrogenase (PoxB). Acetate uptake is mediated either by the low affinity Pta-AckA node or high affinity Acs node, enabling *E. coli* to efficiently scavenge even small amounts of acetate excreted during glucose catabolism [29, 30]. Acetyl-CoA is a major branching point in central metabolism and a precursor for several pathways such as the tricarboxylic acid (TCA) cycle, fatty acid and amino acid synthesis, the glyoxylate bypass and ethanol production [31].

However, *E. coli* is not able to co-utilize glucose and acetate efficiently due to carbon catabolite repression, favoring glucose utilization in the presence of more than one carbon substrate [32, 33]. Furthermore, most *E. coli* strains show acetate secretion upon growth on glucose, a phenomenon usually described as overflow metabolism. Different studies have pointed towards limited respiratory or proteomic capacity of *E. coli* as the potential reason for acetate accumulation [31, 34–36]. Moreover, Acs plays a key role in acetate excretion, as it was reported that *acs* is down-regulated at high specific growth rates [37]. Generally, *acs* is activated by cAMP-CRP, and co-transcribed together with two other genes, a putative inner membrane protein (*yjcH*) and an acetate permease (*actP*) [33]. ActP has previously been described as a cation/acetate symporter, and knock-out strains lacking *actP* grow poorly on acetate as the sole carbon source [38].

Studies using *E. coli* and *Salmonella enterica* have found that in addition to transcriptional control via carbon catabolite repression [31, 33] activity of Acs is also controlled by posttranslational modification. Protein acetyltransferase, *patZ*/Pat, was found to be responsible for acetylation of Acs, rendering the enzyme inactive. In detail, Leu-641 is recognized by Pat, resulting in acetylation of Lys-609 of Acs, and consequently in inactivation of the enzyme [39, 40]. It could be demonstrated that a mutation at Leu-641 in Acs made the enzyme insensitive to acetylation [41]. Acetylation of Acs by Pat can be reversed by NADH-dependent CobB [39, 40]. Generally, *patZ* expression is regulated by cAMP-CRP [42] and during exponential growth phase on glucose *patZ* expression is up-regulated [43]. However, more detailed information on acetylation and activity of Acs is scarcely available, especially in the context of co-utilization of glucose and acetate.

Previous findings showed that *acs* down-regulation during glucose cultivations leads to acetate accumulation [37], deletion of *patZ* leads to more efficient growth on acetate as the sole carbon source in *E. coli* BL 21 [43] and decreased acetate accumulation in glucose limited continuous cultures [44]. To that end, the hypothesis behind the current work was that expression of an acetyl-CoA synthetase insensitive to acetylation (*acs*_L641P) from a constitutive promoter would have a similar effect, enabling efficient co-utilization of glucose and acetate at high concentrations. The aim of this work was to study the effect of overexpression of an acetylation insensitive acetyl-CoA synthetase on a mixed feed system of glucose and acetate in *E. coli* W. To that end, three different strains were constructed, namely ACS_L641P (expressing an acetyl-CoA synthetase insensitive to acetylation from a constitutive promoter), ACS (expressing native acetyl-CoA synthetase from a constitutive promoter) and VC (a control strain carrying an empty vector) which were first characterized in batch cultivations using glucose and acetate, glucose or acetate. The behavior of the strains was further characterized under glucose and acetate-limited conditions using continuous chemostat and accelerostat (A-stat) cultivations. Gene expression analysis during A-stat cultivations using glucose and acetate were performed for acetate metabolism related genes to get insight into the effect of overexpression of an acetyl-CoA synthetase insensitive to acetylation.

## Results

*Escherichia coli* W was chosen for this study because it shows reasonable resistance towards acetate [14], which was also evaluated in batch cultures where growth on up to 2% (w/v) acetate as the sole source of carbon was observed in shake flask cultures, while other *E. coli* strains such as BL21 and K-12 MG1655 did not show growth (data not shown). Sequence comparison of acetyl-CoA synthetase (Acs) from *Salmonella enterica* subsp. *enterica* LT 2 with the enzyme from *Escherichia coli* W revealed that residues Lys-609 and Leu-641 are conserved and the two enzymes show an overall identity of 95% of the amino acids (Additional file 1: Figure S1).

It was previously shown that Lys-609 is the site of acetylation activity by Pat rendering the enzyme inactive. This acetylation can be reversed by NADH-dependent CobB [39, 40]. A random mutation at the residue Leu-641 in Acs made the enzyme insensitive to acetylation, thereby disabling posttranslational modification in presence of high glucose concentrations [41]. To that end, two strains were constructed for expression of either *acs* or *acs*_L641P under control of the constitutive promoter J23114 (Anderson constitutive promotor library).

### Batch cultivations on glucose and acetate

The main hypothesis of this study was that expression of *acs*_L641P from a constitutive promoter should enable *E. coli* W to co-utilize glucose and acetate as both transcriptional and posttranslational control of *acs* by carbon catabolite repression would be circumvented in this case. Additionally, expression of *acs* without the L641P mutation, thus still sensitive to acetylation, from a constitutive promoter was studied. This construct should only be controlled on a transcriptional level but no longer on a posttranslational level.

Batch cultivations on defined media supplemented with 1% (w/v) glucose and 1% (w/v) acetate were carried out with three strains: ACS (strain expressing *acs* from promoter J23114), ACS_L641P (strain expressing *acs*_L641P from promoter J23114) and VC (a strain carrying an empty vector as a control).

Since the aim was to study co-utilization of glucose and acetate, all values mentioned in this paragraph and shown in Tables 1 and 2 are for the exponential phase (cultivation time ~ 4 h until depletion of glucose) where both glucose and acetate were present in the media.

**Table 1:** Growth rate, specific glucose, acetate and base uptake as well as CO_2_ production rates and growth rates for batch processes on glucose + acetate, glucose and acetate during exponential growth phase

	Glc + Ace VC	ACS	ACS_L641P	Glucose VC	ACS_L641P	Acetate VC	ACS_L641P
µ [h^−1^]	0.23 ± 0.05	0.20 ± 0.03	0.27 ± 0.04	0.72 ± 0.01	0.68 ± 0.07	0.19 ± 0.03	0.18 ± 0.04
q_GLC_ [mmol g^−1^ h^−1^]	2.85 ± 0.29	2.71 ± 0.81	3.20 ± 0.38	7.24 ± 0.18	6.90 ± 0.89	–	–
q_ACE_ [mmol g^−1^ h^−1^]	1.76 ± 0.26	1.91 ± 0.58	4.72 ± 0.26	–	–	12.42 ± 2.13	12.36 ± 1.96
q_CO2_ [mmol g^−1^ h^−1^]	8.23 ± 1.96	5.96 ± 2.26	16.33 ± 0.88	16.16 ± 0.11	23.98 ± 0.78	12.79 ± 0.31	17.03 ± 3.32
q_NH3_ [mmol g^−1^ h^−1^]	3.55 ± 0.58	3.29 ± 0.74	1.44 ± 0.86	5.35 ± 0.97	6.30 ± 0.41	–	–

Mean values and errors as standard deviation are displayed

**Table 2:** Yields and carbon recovery for batch processes on glucose + acetate, glucose and acetate, Y_X/S_, Y_CO2/S_, Y_O2/S_

	Glc + Ace VC	ACS	ACS_L641P	Glucose VC	ACS_L641P	Acetate VC	ACS_L641P
Y_X/S_ [Cmol Cmol^−1^]	0.37 ± 0.06	0.37 ± 0.05	0.29 ± 0.03	0.57 ± 0.09	0.44 ± 0.02	0.34 ± 0.01	0.26 ± 0.02
Y_CO2/S_ [Cmol Cmol^−1^]	0.54 ± 0.01	0.56 ± 0.02	0.65 ± 0.04	0.47 ± 0.07	0.57 ± 0.02	0.77 ± 0.02	0.71 ± 0.03
Y_O2/S_ [Omol Cmol^−1^]	0.99 ± 0.16	0.96 ± 0.27	1.26 ± 0.11	0.58 ± 0.13	0.93 ± 0.35	0.85^a^	1.12 ± 0.07
C-recovery [%]	91 ± 7	92 ± 7	94 ± 7	104 ± 16	101 ± 4	111 ± 3	97 ± 5

Mean values and errors as standard deviation are displayed

*Y*_*X/S*_ biomass yield, *Y*_*CO2/S*_ CO_2_ production yield, *Y*_*O2/S*_ oxygen uptake yield

^a^Calculation with only one value, due to O_2_ offgas signal perturbations

**Fig. 1:**
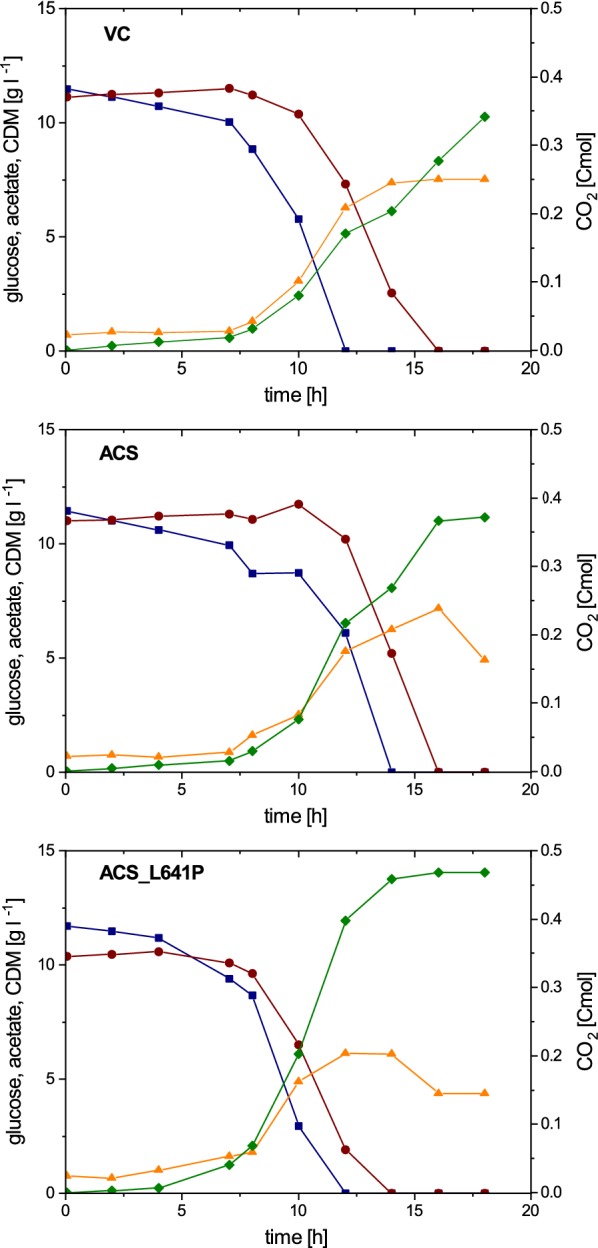
Glucose (blue square), acetate (red circle) and cell dry mass (CDM, orange up-pointing triangle) concentrations as well as accumulated CO_2_ (green diamond) over process time in batches with 1% (w/v) glucose and acetate. Each cultivation was carried out in triplicates. For better visualization, one cultivation is shown as an example

µ, q_GLC_, q_ACE_, q_CO2_, q_NH3_, q_O2_ for the batch cultivations are shown in Table 1, Y_X/S_, Y_CO2/S_, Y_O2/S_, Y_CO2/X_ and the carbon recovery are shown in Table 2. As shown in Fig. 1, all three strains displayed a lag phase of around 4 h. Upon entering exponential growth phase, comparable specific growth and glucose uptake rates for all three strains were observed (Table 1) and at the time glucose was depleted biomass concentrations of 5.71 ± 0.52, 6.22 ± 0.64 and 5.80 ± 0.42 g l^−1^ for ACS_L641P, ACS and the VC, respectively, were observed. At this point the residual acetate concentration for ACS_L641P was significantly lower compared to ACS and VC (3.20 ± 1.23, 7.21 ± 1.74 and 5.20 ± 2.30 g l^−1^, respectively).

Since the biomass concentration was comparable for all strains, a lower acetate concentration at the time point where glucose was depleted conversely indicates that ACS_L641P takes up acetate with a higher specific rate. Indeed, a 2.7-fold increase was observed for q_ACE_ of ACS_L641P, whereas q_ACE_ for ACS remained unchanged compared to VC (Table 1). Interestingly, a similar increase of twofold for q_CO2_ of ACS_L641P was observed, while similar to q_ACE_, the specific carbon dioxide production rate of ACS was comparable to that of VC.

In addition to a higher specific acetate uptake and carbon dioxide production rate, ACS_L641P displayed a 2.5-fold lower specific base consumption rate, indicating that due to higher acetate consumption less ammonia per biomass was required to adjust the pH because of glucose catabolism related acidification.

The different behavior of ACS_L641P with respect to carbon uptake and production compared to ACS and VC can also be observed in the yields at the end of the glucose phase. Compared to the vector control, ACS_L641P showed a 21% decrease in Y_X/S_ while Y_CO2/S_ was increased by 20% (Table 2).

### Batch cultivations on glucose or acetate

To further characterize the effect of expression of acetylation insensitive acetyl-CoA synthetase in *E. coli* W, the behavior of ACS_L641P and VC was studied during cultivations on either glucose or acetate as the sole source of carbon.

The cultivations using glucose as the carbon source showed no significant differences in specific growth and glucose uptake rate for ACS_L641P and VC (Table 1). However, ACS_L641P displayed a 48% increase in q_CO2_, and in addition, showed a 23% decrease in Y_X/S_ and a 21% increase in Y_CO2/S_ (Table 2). These observations may indicate changes in the metabolism of glucose by expression of ACS_L641P.

**Fig. 2:**
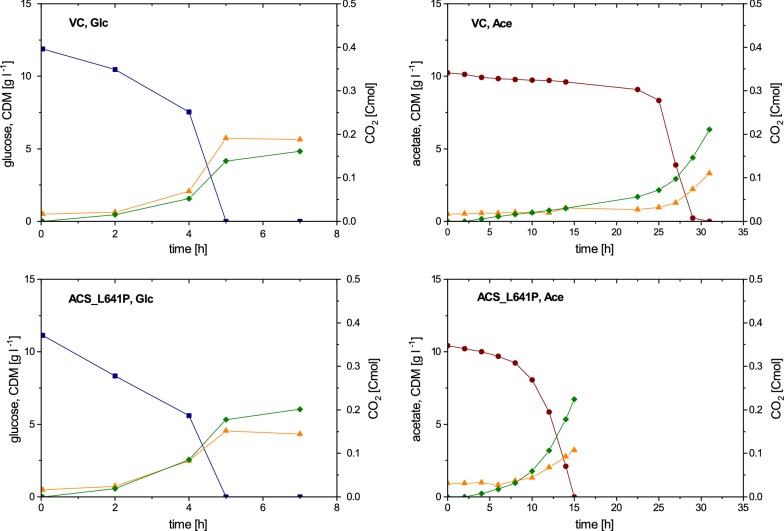
Glucose (blue square), acetate (red circle) and cell dry mass (CDM, orange up-pointing triangle) concentrations as well as accumulated CO_2_ (green diamond) over process time in batches with 1% (w/v) glucose or 1% (w/v) acetate. Each cultivation was carried out in triplicates. For better visualization, one cultivation is shown as an example

For the cultivations using acetate as the carbon source similar values in specific growth and acetate uptake rate for the two strains were observed (Table 1). Despite similar acetate uptake rates, ACS_L641P showed a 33% increase in q_CO2_. Moreover, a significantly longer lag phase and total batch duration was observed for VC compared to ACS_L641P (Fig. 2).

### Continuous cultivations on glucose or glucose and acetate

Based on the findings of the different batch cultivations, a series of continuous cultivations was carried out to study the effect of acetyl-CoA synthetase overexpression under carbon-limited conditions. It is known that upon glucose limitation carbon catabolite repression is less severe, and *acs* expression is induced under these conditions [45]. To that end, the question was if the behavior in C-limited continuous cultures on glucose or co-utilizing glucose and acetate would be different to what has been observed during batch cultures with carbon surplus conditions.

### Chemostat cultivations on glucose

Despite the fact that only small differences were observed for ACS_L641P and VC in batch cultures on glucose, chemostat cultivations at different dilution rates were performed. The aim of this experiment was to study if there were any growth rate dependent effects caused by the expression of *acs*_L641P in the catabolism of glucose observable. Furthermore, it was sought to compare the results obtained for other *E. coli* strains which are less robust against acetate stress. To that end, one chemostat cultivation for each strain was performed at different dilution rates ranging from 0.1 to 0.75 h^−1^ using 2% (w/v) glucose as the carbon source.

**Fig. 3:**
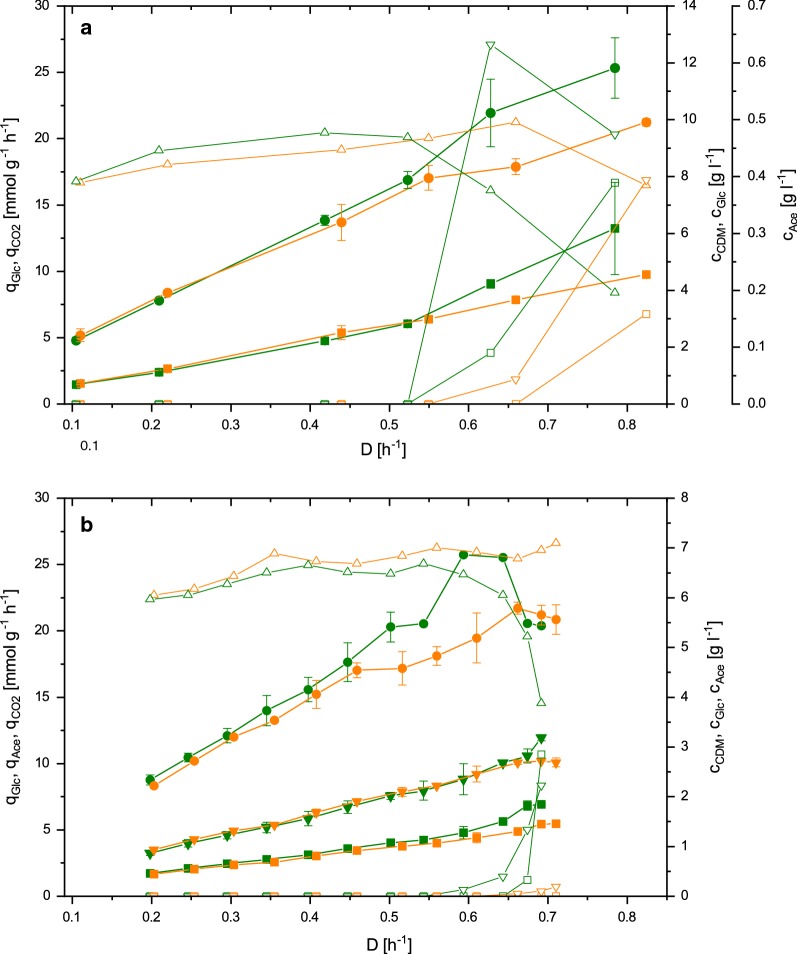
Specific production rates q_GLC_ (filled square), q_ACE_ (filled down-pointing triangle), q_CO2_ (filled circle) of ACS_L641P (green) and the VC (orange) with corresponding glucose (white square), acetate (white down-pointing triangle) and cell dry mass (white up-pointing triangle) concentrations over dilution rate in a glucose chemostat (**a**) and a glucose + acetate mixed-feed A-stat (**b**). Error bars represent the standard deviation of three samples taken during steady state for chemostat cultivations, and the standard deviation of two biological replicates for A-stat cultivations. Due to perturbations in the CO_2_ offgas measurements during the last five samples of the mixed-feed A-stat, q_CO2_ was obtained from a single cultivation, thus no error bars are displayed

As shown in Fig. 3, both, ACS_L641P and VC display similar values for q_GLC_ which is in accordance with the findings for the batch cultures on glucose. However, q_CO2_ for ACS_L641P and VC also showed comparable values for all dilution rates where no acetate or glucose accumulation was observed, which is in contrast to the results of the batch cultures. In detail, both strains displayed an increase in Y_X/S_ with increasing dilution rates, while Y_CO2/S_ decreased (Table 3), i.e. more biomass and less CO_2_ is produced per substrate. Due to this fact, biomass concentrations were 20% higher at dilution rate 0.50 h^−1^ compared to 0.10 h^−1^ for both ACS_L641P and VC. At a dilution rate of 0.63 h^−1^ ACS_L641P started to accumulate acetate and glucose. Upon accumulation of acetate and glucose, q_GLC_ of ACS_L641P increased to higher levels than would be the result of the increased dilution rate. VC started to accumulate acetate at a dilution rate of 0.66 h^−1^, but no glucose accumulation was observed at this growth rate. However, further increasing the dilution rate to 0.82 h^−1^ also led to glucose accumulation for VC.

**Table 3:** Yields for chemostat cultivations on glucose and A-stat cultivations on glucose + acetate, Y_X/S_, Y_CO2/S_

	ACS_L641P		VC	
	Y_X/S_ [Cmol Cmol^−1^]	Y_CO2/S_ [Cmol Cmol^−1^]	Y_X/S_ [Cmol Cmol^−1^]	Y_CO2/S_ [Cmol Cmol^−1^]
Chemostat Glucose				
D [h^−1^]				
0.100	0.455 ± 0.027	0.544 ± 0.023	0.453 ± 0.015	0.557 ± 0.065
0.200	0.562 ± 0.014	0.546 ± 0.001	0.531 ± 0.009	0.528 ± 0.004
0.400	0.562 ± 0.013	0.485 ± 0.007	0.527 ± 0.050	0.425 ± 0.001
0.500	0.553 ± 0.026	0.465 ± 0.005	0.551 ± 0.022	0.445 ± 0.006
0.600	0.443 ± 0.016	0.403 ± 0.034	0.536 ± 0.013	0.379 ± 0.004
0.750	0.393 ± 0.103	0.327 ± 0.057	0.541 ± 0.006	0.363 ± 0.001
A-stat Glucose + acetate				
D [h^−1^]				
0.200	0.451 ± 0.004	0.520 ± 0.042	0.457 ± 0.009	0.489 ± 0.009
0.250	0.458 ± 0.011	0.507 ± 0.042	0.466 ± 0.006	0.488 ± 0.010
0.300	0.474 ± 0.007	0.507 ± 0.044	0.485 ± 0.015	0.500 ± 0.010
0.350	0.492 ± 0.014	0.521 ± 0.072	0.520 ± 0.009	0.506 ± 0.010
0.400	0.503 ± 0.022	0.515 ± 0.068	0.508 ± 0.022	0.493 ± 0.009
0.450	0.492 ± 0.011	0.507 ± 0.068	0.504 ± 0.003	0.488 ± 0.009
0.500	0.490 ± 0.06	0.517 ± 0.037	0.516 ± 0.024	0.447 ± 0.009
0.550	0.505 ± 0.024	0.488^a^	0.528 ± 0.007	0.445 ± 0.009
0.600	0.493 ± 0.041	0.464^a^	0.522 ± 0.037	0.433 ± 0.008
0.650	0.458 ± 0.015	0.469^a^	0.514 ± 0.004	0.440 ± 0.009
0.680	0.416 ± 0.015	0.410^a^	0.501 ± 0.001	0.400 ± 0.011
0.700	0.405 ± 0.012	0.414^a^	0.514 ± 0.012	0.393 ± 0.009

Mean values and errors as standard deviation are displayed

*Y*_*X/S*_ biomass yield, *Y*_*CO2/S*_ CO_2_ production yield

^a^Calculation with only one value, due to CO_2_ offgas signal perturbations

### A-stat cultivations on glucose and acetate

During batch characterization, increased specific acetate uptake rates for ACS_L641P were obtained when glucose and acetate were co-utilized. Based on this finding, it was hypothesized that ACS_L641P should be able to co-utilize glucose and acetate more efficiently compared to VC also in continuous cultures co-utilizing both substrates. To investigate this hypothesis, accelerostat (A-stat) cultivations (continuous cultures with a constantly increasing dilution rate) [46] starting at a dilution rate of 0.20 h^−1^ were performed. The dilution rate was increased at a rate of 0.01 h^−2^ until a dilution rate of 0.70 h^−1^ using 1% (w/v) glucose and 0.5% (w/v) acetate as carbon sources. Based on the batch cultures, it was speculated that ACS_L641P would accumulate acetate at higher dilution rates compared to VC as higher dilution rates in C-limited cultures with constant biomass concentrations correspond to higher specific substrate uptake rates.

For both strains, the specific rates q_GLC_, q_ACE_ and q_CO2_ as well as biomass concentrations constantly increased with increasing dilution rate until cell wash out started to occur (Fig. 3b). Acetate accumulation in ACS_L641P and VC started at a dilution rate of 0.59 and 0.66 h^−1^, respectively. Glucose was accumulated at 0.67 h^−1^ in ACS_L641P, whereas no glucose accumulation was observed for VC until the end of the experiment (D = 0.71 h^−1^).

With respect to Y_X/S_ and Y_CO2/S_, a similar behavior as for the chemostat cultivations with glucose as the carbon source was observed. Specifically, both strains display a shift from CO_2_ to biomass at high dilution rates, resulting in 15% increased biomass yield at a dilution rate of 0.55 h^−1^ compared to the initial dilution rate of 0.20 h^−1^ for the VC. The ratio between biomass and CO_2_ production for ACS_L641P did not significantly change as a function of the dilution rate. Upon glucose and acetate accumulation Y_X/S_ and Y_CO2/S_ sharply decrease due to reduced carbon source consumption for ACS_L641P. However, for VC only the decrease of Y_CO2/S_ could be observed upon accumulation of acetate, while Y_X/S_ did not decrease.

To further investigate the performance of the two strains, ACS_L641P and VC, gene expression analysis was performed for several genes of the acetate metabolism. Two dilution rates were investigated, 0.20 and 0.65 h^−1^. Since the outcome of the experiment did not confirm the hypothesis that ACS_L641P should be able to more efficiently co-utilize glucose and acetate at high dilution rates (corresponding to high specific uptake rates) gene expression analysis might be able to shed light on what might be the reason for the observed behavior of the two strains.

**Fig. 4:**
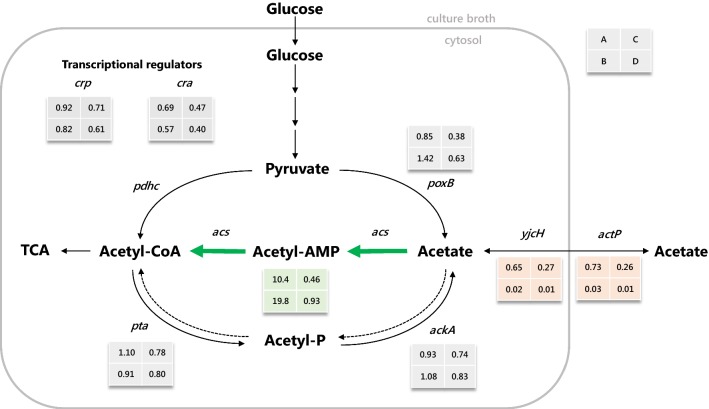
Acetate metabolism in *E. coli* and results of the gene expression analysis. Green arrows indicate overexpression of *acs* with Leu-641 residue changed to proline under control of a constitutive promoter. Solid black lines show glucose catabolism and acetate formation pathways, dashed lines indicate reverse reactions. Grey boxes show fold change of expression levels: A, ACS_L641P vs. VC at dilution rate 0.2 h^−1^, B, ACS_L641P vs. VC at dilution rate 0.65 h^−1^, C, 0.65 h^−1^ vs. 0.2 h^−1^ for VC, D, 0.65 h^−1^ vs. 0.2 h^−1^ for ACS_L641P. *acs*, acetyl-CoA synthetase, *phdc*, pyruvate dehydrogenase complex, *poxB*, pyruvate oxidase, *pta*, phosphotransacetylase, *ackA*, acetate kinase, *yjcH*, putate membrane protein, *actP*, acetate permease, *cra*, catabolite repressor activator, *crp*, cAMP receptor protein. The fold changes given represent the mean of two biological and three technical replicates. For visualization reasons, standard errors and p-values are not shown, but can be seen in Additional file 2: Table S1

The expression levels of the eight genes investigated were each compared between different dilution rates (e.g. vector control at 0.20 h^−1^ vs. 0.65 h^−1^) as well as between strains (e.g. VC vs. ACS_L641P at D = 0.65 h^−1^). Figure 4 shows the results of the gene expression analysis depicted in a simplified metabolic network (standard errors and *p* value are given in Additional file 2: Table S1).

Most prominently, ACS_L641P showed 10.4- and 19.8-fold increased expression levels for *acs* at dilution rates 0.20 and 0.65 h^−1^, respectively, compared to VC. Furthermore, the expression level of *acs*_L641P in ACS_L641P did not significantly decrease upon increased dilution rates, while a twofold downregulation for *acs* was observed in VC. Hence, the apparently increased relative expression level of *acs* in ACS_L641P at 0.65 h^−1^ compared to the VC is a consequence of down-regulation of *acs* in the VC and seems to be unrelated to the acetate concentration or dilution rate. At a dilution rate of 0.65 h^−1^, a sharp downregulation of *yjcH* and *actP* was observed for ACS_L641P compared to VC (less than 5% of original expression level). Generally, expression levels of *yjcH* and *actP* dropped significantly when comparing dilution rate 0.65 h^−1^ to the initial dilution rate of 0.20 h^−1^ for both strains (~ fourfold decrease for VC at 0.65 h^−1^ vs. 0.20 h^−1^ and < 5% expression level for ACS_L641P at 0.65 h^−1^ vs. 0.20 h^−1^). Genes involved in acetate metabolism (*pta*, *ackA*, *poxB*) showed lower expression levels at high dilution rates for both strains, although for *poxB* the decrease in expression rate at high dilution rates was less severe for ACS_L641P compared to VC.

The expression levels for the transcriptional regulators *crp* and *cra* decreased with increasing dilution rates, with the effect being slightly more prominent for ACS_L641P than for VC (Fig. 4).

## Discussion

The aim of the present study was to investigate if and how efficient co-utilization of glucose and acetate in *E. coli* can be achieved. To that end, it was studied how expression of an acetylation insensitive acetyl-CoA synthetase from a constitutive promoter affects co-utilization of glucose and acetate in *E. coli* W, both under high carbon conditions in batch cultures and carbon-limiting conditions of continuous cultures.

During aerobic batch cultivations using glucose and acetate as carbon sources it was found that the strain expressing *acs*_L641P displayed a 2.7-fold increased specific acetate uptake rate, whereas no change in q_ACE_ was observed for ACS compared to VC. These findings seem to confirm the hypothesis that activity of Acs alone during metabolism of high concentrations of glucose is sufficient to enable more efficient co-utilization of acetate. It would appear that under high glucose and acetate concentrations, where only Pta-AckA but not Acs are active, expression of *acs* from a constitutive promoter and insensitive to acetylation is sufficient to partially overcome control mechanisms by glucose-mediated carbon catabolite repression, thus presenting a proof-of-principle. However, acetate uptake for VC and ACS are quite significant. This could potentially be explained by previous reports showing that *E. coli* can both produce and assimilate acetate during glucose metabolism via Pta-AckA, and that the direction of the pathway depends only on extracellular concentrations of acetate [33].

Another phenomenon observed for ACS_L641P during all cultivations using glucose and acetate was an approximate 20% increase in Y_CO2/S_ and therefore lower Y_X/S_, i.e. more CO_2_ and less biomass were produced in ACS_L641P compared to ACS and VC.

Steady energy input for gene expression and protein production [37] was ruled out as the reason for this shift in yields, as the comparison of ACS and ACS_L641P showed that ACS did not display the same shift.

Therefore, two other explanations can be argued to be responsible for the different behavior of ACS_L641P, namely either energy requirements by the activity of Acs in ACS_L641P or a different metabolic flux pattern.

Regarding energy, it can be stated that the net consumption of the Pta-AckA-Acs cycle is 1 ATP (2 ATP utilized by Acs, 1 ATP produced by Pta-AckA) [33, 37]. If simultaneous assimilation and dissimilation is assumed entirely through this cycle, 16% of the overall ATP needs of a cell would be required for the recycling of acetyl-CoA [33, 47]. Increasing the activity of Acs in ACS_L641P by overexpression would likely result in a higher overall activity of the Pta-AckA-Acs cycle. As little or no activity of *acs* would be expected in either ACS or VC, this enhanced Pta-AckA-Acs cycle activity in ACS_L641P would require more energy, which in turn would not be available for biomass formation, thus lowering Y_X/S_ and increasing Y_CO2/S_. This is further supported by a previous report that during exponential growth on glucose, *patZ*, the gene coding for protein acetyltransferase (Pat) is expressed at high levels, thus resulting in Acs acetylation and inactivation [43] and therefore only ACS_L641P would display Acs activity but not VC and ACS.

Regarding a different metabolic flux pattern, metabolic flux analysis showed that *E. coli* shows little glyoxylate cycle activity during glucose metabolism, while high fluxes through the glyoxylate shunt and only small fluxes for the TCA cycle were observed during metabolism of acetate [48]. The anaplerotic glyoxylate cycle negatively controlled by isocitrate lyase regulator (IclR) during glucose excess [34]. As a consequence, the glyoxylate shunt is likely to be inactive during co-utilization of glucose and acetate. Strain ACS_L641P catabolizes acetate to a higher extent in the presence of glucose compared to ACS and VC. This additional acetate would therefore be channeled into the TCA cycle rather than the glyoxylate cycle, resulting in a phenotype producing more CO_2_ (2 mol CO_2_ in the TCA cycle compared to no CO_2_ in the glyoxylate cycle).

When grown on acetate as sole carbon source, the lag phase of VC was eightfold longer than that of ACS_L641P, thus resulting in an overall increased total batch duration. However, µ as well as q_ACE_ are not significantly different in the exponential phase for both strains. The long lag-phase might be caused by the toxicity of acetate as a weak acid, causing a decrease in intracellular pH, an increase in osmotic pressure and interference with methionine biosynthesis [21, 49–52]. ACS_L641P is likely to overcome acetate toxicity by more efficient consumption due to overexpression of *acs*_L641P compared to VC [31]. Increased growth on acetate was also shown in an *E. coli* BL21 *patZ* knock-out strain [43], in both cases likely due to more active Acs (i.e. non-acetylated). When acetate is used as the sole carbon source, *acs* should not be repressed by carbon catabolite repression and thus expression rates in ACS_L641P and VC are expected to be more similar compared to mixed substrate fermentations, where *acs* is repressed in the VC. This fact can explain that there is no significant difference in q_ACE_, which also corresponds to what has been observed previously [27]. Acetate consumption might also be limited by transport or subsequent metabolic reactions, which is further supported by the fact that inactivation of i*clR* increased acetate consumption in *E. coli* [27].

It was reported that *E. coli* W shows higher growth rates on acetate compared to other *E. coli* strains (BL21-DE3, K-12 W3110, and K-12 MG1655), and that protein acetylation of Acs by Pat is likely strain specific, as different expression levels for *patZ* were observed for *E. coli* BL21 and a K-12 strain in glucose batch cultivations [43]. In cultivations with 10 g l^−1^ acetate, a growth rate of 0.46 h^−1^ and a specific acetate uptake rate of 3.66 mmol g^−1^ h^−1^ were reached [27]. In this study, lower growth rates and higher consumption rates of 0.19 h^−1^ and 12.4 mmol g^−1^ h^−1^, respectively, were observed.

Only very low amounts of acetate (less than 0.5 g l^−1^) accumulated at the end of the aerobic batches on glucose in ACS_L641P and VC, which corresponds well with previous reports for *E. coli* W describing a highly oxidative metabolism [14] and represents a distinct difference to other strains accumulating higher amounts of acetate such as K-12 BW25113 [31].

Chemostat cultivations on glucose with ACS_L641P and VC showed ambiguous results for the two strains. In ACS_L641P, accumulation of acetate and cell wash out (D = 0.63 h^−1^) occurred almost simultaneously and at lower dilution rates compared to VC. This observation is different to previous reports where A-stat cultivations on glucose with *E. coli* K-12 MG1655 showed acetate accumulation between growth rates of 0.27 and 0.54 h^−1^, and above the latter threshold, glucose was accumulated and cells were washed out [37]. In the present study, the phase of acetate accumulation was much shorter and both accumulation as well as wash out were observed at higher dilution rates.

Considering that cell wash out started to occur in ACS_L641P at a dilution rate similar to the mean specific growth rate observed during batch cultures (0.68 h^−1^ vs 0.72 h^−1^ for ACS_L641P and VC, respectively), these findings are somewhat surprising. During glucose metabolism, the Pta-AckA-Acs cycle is thought to be responsible for balancing of the intracellular acetyl-CoA and acetyl-P pools [31]. Natural imbalance of the cycle at the expense of Acs results in accumulation of acetate during glucose excess cultures [45]. It was speculated that higher expression levels of *acs*_L641P in ACS_L641P, creating an imbalance in favor of Acs in comparison to the other genes of the Pta-AckA-Acs cycle would possibly enable ACS_L641P to more efficiently cycle acetate, thus leading to delayed accumulation of acetate compared to the VC. This hypothesis is supported by previous reports where the coordinated activation of Acs by inactivation of Pat and the TCA cycle by deletion of *arcA* led to a delayed onset of overflow metabolism and an overall significantly decreased accumulation of acetate in accelerostat cultures using glucose [44].

As the findings were contrary to this hypothesis, accumulation of acetate at lower dilution rates in ACS_L641P could potentially be a consequence of the lack of additional TCA cycle activity an *arcA* knock-out strain would display. Furthermore, earlier onset of acetate accumulation could be due to increased energy dissipation as a result of higher Pta-AckA-Acs cycle activity owing to overexpression of *acs*_L641P in ACS_L641P.

The latter could also explain what was observed for the A-stat cultivations co-utilizing glucose and acetate. Similar to the glucose chemostats, acetate accumulation in A-stats occurred at lower dilution rates for ACS_L641P compared to VC. Moreover, Y_X/S_ and Y_CO2/S_ did not differ significantly for both strains, which is in contrast to the results obtained from the batch cultures on glucose and acetate.

However, it was reported that compared to glucose surplus batch cultivations during glucose limited chemostat cultivations transcription of *acs* is up-regulated [43, 45]. As a consequence, a more similar behavior for ACS_L641P and VC compared to batch cultures on glucose and acetate appears reasonable at low specific acetate uptake rates, while it was assumed that additional Acs activity by overexpression in ACS_L641P would allow for more efficient acetate uptake (higher q_ACE_) at high dilution rates.

Despite this assumption, earlier acetate accumulation and cell wash out for ACS_L641P compared to VC in glucose-acetate A-stat cultivations could have been caused by a severe imbalance of the Pta-AckA-Acs cycle due to *acs*_L641P overexpression. In cultures co-utilizing glucose and acetate, both the Pta-AckA node as well as Acs could be responsible for acetate uptake, where 1 or 2 mol ATP per mol of acetate would be required for uptake, respectively.

Based on the results of the gene expression analysis, the ratio between *acs* and *pta*-*ackA* in ACS_L641P is much higher compared to VC. Hence, acetate flux via Acs rather than the Pta-AckA node could occur already at lower dilution rates for ACS_L641P compared to VC, resulting in higher ATP consumption for acetate uptake.

Another interesting finding of the gene expression analysis was that *yjcH* and *actP* were significantly down-regulated at higher dilution rates. This effect was more severe for ACS_L641P than for VC, and could potentially be the reason for earlier acetate accumulation in ACS_L641P, if acetate transport at high dilution rates is less effective or limiting. To shed light on this, flux analysis using labeled acetate could be used to determine the source of acetate accumulation (feed medium vs. excretion of intracellular acetate). Additionally, overexpression of *actP* could help to uncover transport limitations.

Finally, cell wash out and acetate accumulation in ACS_L641P could be caused by energy demand for gene expression and protein production compared to VC, which would be expected to be more severe at high dilution rates.

However, it must be emphasized that in this study *E. coli* W was shown to be naturally very efficient in co-utilization of glucose and acetate, and that the strategy pursued here could have led to different results in notorious acetate excreting *E. coli* strains.

## Conclusion

In this study it was shown that *E. coli* W is a promising candidate for processes relying on efficient acetate uptake or low acetate excretion. In detail, the overexpression of an acetylation-insensitive acetyl-CoA synthetase, for the first time significantly increased (2.7-fold) the specific acetate uptake rate in a mixed batch system using glucose together with high concentrations of acetate. Additionally, shorter batch durations during cultures using high concentrations of acetate were observed for the overexpression strain, likely due to *acs* related alleviation of acetate toxicity. Further characterization in chemostat and A-stat cultures showed that *E. coli* W is naturally capable of efficiently co-utilizing glucose and acetate in C-limited A-stat cultivations as no significant differences were found between the overexpression strain and a control strain with respect to acetate uptake. To that end, further work is required to gain a deeper understanding of metabolism in continuous cultures co-utilizing glucose and acetate. Metabolic flux analysis could shed light on the intracellular fluxes for glucose and acetate and help identify targets for further engineering. Among others, acetate transport could be manipulated by overexpression of *actP* for enhanced acetate uptake or genome engineering to deregulate the TCA cycle (via deletion of *arcA*) and glyoxylate cycle (via deletion of *iclR*) could further improve co-utilization of glucose and acetate in *E. coli* W.

## Methods

### Bacterial strains and media

*Escherichia coli* W (DSM 1116 = ATCC 9637 = NCIMB 8666) was obtained from DSMZ (Braunschweig, Germany) and used for all cultivations in this study. *Escherichia coli* BL21 (DE3) was obtained from New England Biolabs (MA, USA) and used as host for plasmid assembly and propagation.

Lysogeny broth (LB) containing per litre liquid medium: soy peptone, 10 g, yeast extract, 5 g, sodium chloride, 10 g, and LB agar additionally containing per litre: agar agar, 15 g, was used for all cloning and plasmid propagation steps. 2 × LB medium was used for all pre-cultures (soy peptone and yeast extract concentration doubled).

For all bioreactor cultivations defined medium containing per litre: KH_2_PO_4_, 13.3 g, (NH_4_)_2_HPO_4_, 4.00 g, citric acid, 1.70 g, MgSO_4_ * 7H_2_0, 1.2 g, Fe(III)citrate, 0.100 g, EDTA, 0.0084 g, Zn(CH_3_COO)_2_ * 2 H_2_O, 0.013 g, CoCl_2_ * 6 H_2_O, 0.0025 g, MnCl_2_ * 4 H_2_O, 0.015 g, CuCl_2_ * 2 H_2_O, 0.0012 g, H_3_BO_3_, 0.0030 g, Na_2_MoO_4_ * 2 H_2_O, 0.0025 g as described previously was used. As carbon source either 1% (w/v) glucose + 1% (w/v) acetate, 1% (w/v) glucose or 1% (w/v) acetate was used. The medium for the continuous process was equivalent to the batch medium and contained either 2% (w/v) glucose or 1% (w/v) glucose + 0.5% (w/v) acetate. For the continuous culture with glucose and acetate as carbon sources, 3.24 g l^−1^ NH_4_Cl were added to the feed medium.

Liquid and solid media were supplemented with 50 µg ml^−1^ kanamycin or 100 µg ml^−1^ ampicillin as necessary.

### Plasmid and strain construction

The *acs* gene coding for acetyl-CoA synthetase was PCR amplified from genomic DNA of *E. coli* W using Q5 High-Fidelity DNA Polymerase (New England Biolabs, MA, USA) and primers FS2_acs_fw and FS3_acs_rev (Table 4). All primers in this study were purchased from Integrated DNA Technologies (IA, USA). To introduce the L641P mutation into acs and to add the fusion sites (FS) required for GoldenMOCS cloning, two PCR reactions amplified *acs* until position 641 using primers acs_fw and ACS_L641P_rev. In a second PCR reaction, FS sites and the rest of the coding sequence was added using primers FS2_acs_fw and FS3_acs_L641P_rev.

**Table 4:** List of used primers in this work

Name	Sequence (5′-3′)
acs_fw	atgAGCCAAATTCACAAACAC
acs_L641P_rev	CTTCAAGCGGCTTCTC
FS2_acs_fw	GATCGGTCTCACatgAGCCAAATTCACAAACAC
FS3_acs_rev	GATCGGTCTCAAAGCttaCGATGGCATCGCG
FS3_acs_L641P_rev	GATCGGTCTCAAAGCttaCGATGGCATCGCGATAGCCTGCTTCTCTTCAAGCGGCTTCTC
seq_fw	GCAGTCCAGTTACGCTG
seq_rev	CGTGGACCGATCATACG
acs_seq_in_fw	GCAGTATTCCGCTGAAG
acs_seq_in_rev	GGTAGCGCCTTCCAG

Inserted mutations are underlined

For all cloning steps in this study GoldenMOCS, a Golden Gate based cloning system, was used [53, 54]. The two PCR fragments were used for assembly into backbone 1 (BB1) of the GoldenMOCS as described previously and clones were verified for correct assembly and PCR amplification via restriction digests and Sanger sequencing (Microsynth AG, Switzerland) using primers seq_fw and seq_rev, respectively (Table 4).

BB2 assembly was used to arrange *acs*/*acs*_L641P in a single expression cassette under control of the constitutive promoters BBa_J23114 (114p) of the Anderson promoter library and BBa_B1001 as terminator (Table 5).

**Table 5:** Generated plasmids and used strains in this work

Name	Source
Plasmids	
BB1_pIDTSmart(Kan^R^)_FS1_114p_FS2	Sarkari et al. [54]
BB1_pIDTSmart(Kan^R^)_FS2_amilCP_FS3	Sarkari et al. [54]
BB1_pIDTSmart(Kan^R^)_FS3_BBa_B1001_FS4	Sarkari et al. [54]
BB1_pIDTSmart(Kan^R^)_FS2_acs_FS3	This work
BB1_pIDTSmart(Kan^R^)_FS2_acs_L641P_FS3	This work
BB2_pUC(Amp^R^)_LinkerA_FS1_FS4_LinkerB	Sarkari et al. [54]
BB2_pUC(Amp^R^)_LinkerA_114p_acs_LinkerB	This work
BB2_pUC(Amp^R^)_LinkerA_114p_acs_L641P_LinkerB	This work
BB3_pUC(Kan^R^)_LinkerAD	Sarkari et al. [54]
BB3_pUC(Kan^R^)_LinkerA_114p_acs_BBa_B1001_LinkerB	This work
BB3_pUC(Kan^R^)_LinkerA_114p_acs_L641P_BBa_B1001_LinkerB	This work
Strains	
*Escherichia coli* BL21 (DE3)	New England Biolabs
*Escherichia coli* W DSM 1116	DSMZ

*BB* backbone; *114p* BBa_J23114, constitutive promoter from Anderson promoter library; *FS* fusion site; *BBa_B1001* artificial terminator

BB3 assemblies were carried out to change the antibiotic resistance cassette to kanamycin (Table 5). All BB2 and BB3 plasmids were checked for correct assembly by restriction digests.

BB3 plasmids carrying either a functional *acs*/*acs*_L641P cassette or an empty BB3 were transformed into chemically competent *E. coli* W using the heat shock method.

### Preculture preparation

Glycerol stocks (stored at − 80 °C in 10% (w/v) glycerol) were streaked onto LB agar plates containing 50 µg ml^−1^ kanamycin and incubated overnight at 37 °C. Subsequently, 250 ml LB medium was inoculated with a single colony and incubated in 1 l shake flasks for 14 h at 37 °C and 200 rpm. The cells were grown until they reached an OD_600_ of ~ 4, pelleted and washed twice with 80 ml sterile, 0.9% (w/v) NaCl solution (4800 rpm, 30 min, room temperature) and resuspended in 20 ml 0.9% (w/v) NaCl solution. The OD_600_ of the resuspended culture was determined and a volume appropriate to inoculate the bioreactor with an OD_600_ of 1 (corresponding to a CDW of approx. 0.59 g l^−1^) was transferred to the bioreactor.

### Bioreactor cultivations

Batch cultivations were performed in four parallel DASGIP Benchtop Bioreactors for Microbiology (Eppendorf AG, Hamburg, Germany) with an initial OD_600_ of 1 and an initial batch volume of 1 l. The temperature for all cultivations was 37 °C. To maintain aerobic cultivation conditions all reactors were stirred with 1400 rpm and gassed continuously with pressurized air at 2 vvm (= 120 l h^−1^). The dissolved oxygen concentration was monitored using a VisiFerm DO 225 (Hamilton, Reno, NV, USA) and remained above 30% throughout all cultivations. A pH electrode (Mettler-Toledo GmbH, Giessen, Germany) was used for monitoring the pH value and a constant pH of 7 was maintained by addition of NH_4_OH (12.5% v/v) and 5 M HCl. CO_2_ and O_2_ concentrations were measured using the off-gas analysis module GA4 Eppendorf AG, Hamburg, Germany). Samples were taken immediately after inoculation, then at least every 2 h during batch phases as well as directly after the observed phase and batch end.

For the continuous culture 200 ml medium was inoculated with an OD_600_ of 1 in four parallel DASBOX Mini Bioreactors (Eppendorf AG, Hamburg, Germany). The reactors were stirred with 1400 rpm; the pH was set to 6.8 (to avoid media precipitation) and measured by a pH electrode (Mettler-Toledo GmbH, Giessen, Germany). NH_4_OH (12.5% v/v) and 5 M HCl were added to correct the pH. To assure aerobic cultivation conditions, air was added at 2 vvm (= 24 l h^−1^) and the dissolved oxygen concentration, which was monitored by a VisiFerm DO 225 probe (Hamilton, Reno, NV, USA), was kept above 30% by the addition of pure oxygen. Offgas analysis (CO_2_ and O_2_ concentrations) was carried out using the off-gas analysis module GA4 (Eppendorf AG, Hamburg, Germany).

For chemostat cultures, feed medium with 2% (w/v) glucose was used and dilution rates of 0.10, 0.20, 0.40, 0.50, 0.60 and 0.75 h^−1^ were tested. After three volume changes, at least three samples were taken with a minimum interval of 2 h between samples. The average of these triplicates was used for all further calculations.

In the accelerostat (A-stat), feed medium with 1% (w/v) glucose and 0.5% (w/v) acetate was used. After the initial batch, the dilution rate was set to 0.20 h^−1^ (F = 40 ml h^−1^). After more than three volume changes (= 15 h), a steady state was assumed and a sample was taken. Subsequently, the dilution rate was increased linearly with 0.01 h^−2^ (2 ml h^−2^) and samples were taken every five hours until the dilution rate reached 0.70 h^−1^.

### Biomass determination

Samples from bioreactor cultivations taken at regular intervals were used for gravimetric determination of the cell dry weight (CDW) (in triplicate for batch, duplicates for chemostat and A-stat cultures). Briefly, 4 ml culture broth was centrifuged (4500 rpm, 10 min, 4 °C) and washed with deionized water in pre-weighed test glasses. The pellet was dried for at least 72 h at 105 °C. OD_600_ was measured in a spectrophotometer (Genesys™ 20, Thermo Scientific, Waltham, Massachusetts, USA) against a blank of water.

### HPLC analysis

The substrate and metabolite concentrations of the culture broth were measured by HPLC with an Agilent system (1100 series, Agilent Technologies, Santa Clara/CA, USA) using an Aminex HPX-87H column (300 × 7.8 mm, Bio-Rad, Hercules/CA, USA) with a refractive index detector (Agilent 1100 series G1362A, Agilent Technologies, Santa Clara/CA, USA) and an UV detector (Agilent 1100 series G1315A, Agilent Technologies, Santa Clara/CA, USA). The column was operated at 60 °C with a flow of 0.6 ml min^−1^ for 30 min and with 4 mM H_2_SO_4_ as a mobile phase. The HPLC run was controlled and monitored using ChemStation for LC 3D systems (Agilent Technologies, Santa Clara/CA, USA). For sample preparation, 450 µl cell-free supernatant was mixed with 50 µl 40 mM H_2_SO_4_ and 10 µl sample was injected for analysis. 5-point calibration curves treated in the same way as the samples were used to determine substrate and metabolite concentrations in the samples.

### Biomass composition

To determine biomass composition *E. coli* W was grown for 7 h at 37 °C and 200 rpm in defined medium supplemented with 1% (w/v) glucose. Cells were pelleted (4500 rpm, 30 min, 4 °C) and washed three times with sterile filtered, deionized water, transferred to 50 ml tubes and lyophilized at − 55 °C and 0.02 mbar (Martin Christ, alpha 1–4 LD plus, Osterode am Harz, Germany) for 24 h. The pellet was subsequently milled and biomass composition with respect to carbon, hydrogen, nitrogen, oxygen, phosphorous and sulphur was determined in triplicate (University of Vienna, Vienna, Austria). From the results the elementary composition of the biomass was determined to be C_1.000_H_1.676_O_0.439_N_0.234_P_0.018_S_0.005_, i.e. the carbon content of *E. coli* W dry biomass is 46.1% (w/w).

### Gene expression analysis

Immediately after samples (at 0.2 and 0.65 h^−1^ for ACS_L641P and VC) were taken from A-stat cultivations, 100 µl samples were aliquoted and centrifuged in a desk centrifuge for 30 s, 16,000*g* at 4 °C. The supernatant was discarded and the cell pellet was snap frozen in liquid nitrogen. The samples were stored at − 80 °C until further use.

RNA from frozen sample was isolated using the PureLink RNA Mini Kit (Ambion by life technologies, ThermoFisher Scientific, USA) according to the manufacturer’s recommendation. RNA was eluted in RNase free MQ water. Subsequently, genomic DNA was digested using RNAse free DNAse (ThermoFisher Scientific, USA) together with RiboLock RNase inhibitor (ThermoFisher Scientific, USA) in a 20 µl reaction, using 2 µl of purified RNA. The DNA-free purified RNA was quantified using a Nanodrop 1000 (ThermoFisher Scientific, USA).

The RNA was reverse transcribed using the RevertAid H Minus First Strand cDNA kit (ThermoFisher Scientific, USA) according to the manufacturer’s protocol using random hexamer primers (20 µl reaction volume).

Gene expression levels were determined by gene-specific quantitative real-time PCR using Luna Universal qPCR Master Mix (New England Biolabs, USA). The primers for the qPCR were designed using the PrimerQuest tool (Integrated DNA Technologies, USA) and are listed in Additional file 2: Table S3. The genes for a 16S ribosomal rRNA gene, *rrsG*, and a DNA replication terminus site-binding protein, *tus*, were used as housekeeping genes for normalization. The qPCR reaction was performed on a qTower 2.2 (Analytik Jena AG, Germany) system using the program specified in Additional file 3. Determination of primer efficiency was performed by establishing a standard curve from a dilution series of cDNA (dilution steps 5, 10, 20, 50 and 100) for the housekeeping genes *rrsG* and *tus*. For the individual genes, each qPCR reaction was performed in triplicates for each condition.

Data evaluation was performed as described previously [55]. In brief, the mean C_t_ values were determined by calculating the average of the triplicate measurements for each gene and condition. The ΔC_t_ values were calculated by subtracting the average mean C_t_ value of the two housekeeping genes from the mean C_t_ value of the gene of interest. ΔΔC_t_ is constituted by the difference between the C_t_ value of the sample of interest (ACS_L641P at 0.2 and 0.65 h^−1^, respectively, and VC at 0.65 h^−1^) and the reference sample (VC at 0.2 h^−1^). The relative fold changes shown were calculated by averaging the fold changes of the two biological replicates using Relative quantity = 2 − ΔΔC_t_. The deviation given in Additional file 2: Table S1 is the standard error of the two biological and three technical replicates.

### Data evaluation

Data were analyzed according to Additional file 3.

## Additional files

**Additional file 1: Figure S1.** Sequence alignment of Acs of E. coli W and S. enterica LT 2. Residue Lys-609 highlighted by green box represents site of acetylation by Pat, residue Lys-641 highlighted by red box indicates recognition site of Pat for acetylation.

**Additional file 2: Table S1.** Mean fold change values including standard errors for the four comparisons A, B, C, D (named in the same way as in Figure 1 of the manuscript). Values highlighted in green represent significantly different expression levels (p-value 0.05). **Table S2.** qPCR program used for gene expression analysis. Lid temperature was set to 95 °C. **Table S3.** List of primers for gene expression analysis.

**Additional file 3.** Statistical/data evaluation.

## Abbreviations

Ra_inert_: inert gas ratio (–)
y: mole fraction (–)
y_wet_: O_2_ concentration in off-gas diluted by water content (without bioreaction) (–)
${\text{ex}}_{{{\text{H}}_{2} {\text{o}}}}$: water content in off-gas (–)
V_m_: molar volume of gas at norm conditions (0 °C and 1 atm) (nl mol^−1^)
x: concentration of biomass in the fermentation broth (mmol l^−1^)
s: concentration of substrate in the fermentation broth (mmol l^−1^)
X: total amount of biomass in the fermentation broth and sampling (Cmol)
S: total amount of substrate in the fermentation broth and sampling (Cmol)
${\text{n}}_{{{\text{CO}}_{2} }}$: accumulated total amount of carbon dioxide (Cmol)
${\text{n}}_{{{\text{O}}_{2} }}$: accumulated total amount of oxygen (Omol)
n_c,i_: amount of mole carbon from component i (Cmol)
t: time (h)
${\text{q}}_{{{\text{CO}}_{ 2} }}$: specific CO_2_ production rate (mmol g^−1^ h^−1^)
${\text{q}}_{{{\text{NH}}_{ 3} }}$: specific base consumption rate (mmol g^−1^ h^−1^)
r_C_,_i_: volumetric uptake/production rate (Cmol l^−1^ h^−1^)
Y_i/S_: subtrate yield (Cmol Cmol^−1^)
q_C_,_i_: specific uptake/production rate (Cmol Cmol^−1^ h^−1^)

## References

[1] Wurm DJ, Quehenberger J, Mildner J, Eggenreich B, Slouka C, Schwaighofer A (2018). Teaching an old pET new tricks: tuning of inclusion body formation and properties by a mixed feed system in *E. coli*. Appl Microbiol Biotechnol.

[2] Eggenreich B, Willim M, Wurm DJ, Herwig C, Spadiut O (2016). Production strategies for active heme-containing peroxidases from *E. coli* inclusion bodies—a review. Biotechnol Rep Amst Neth.

[3] Ferrer-Miralles N, Saccardo P, Corchero JL, Xu Z, García-Fruitós E (2015). General introduction: recombinant protein production and purification of insoluble proteins. Methods Mol Biol Clifton NJ.

[4] Ohta K, Beall DS, Mejia JP, Shanmugam KT, Ingram LO (1991). Genetic improvement of *Escherichia coli* for ethanol production: chromosomal integration of Zymomonas mobilis genes encoding pyruvate decarboxylase and alcohol dehydrogenase II. Appl Environ Microbiol.

[5] Zhou S, Iverson AG, Grayburn WS (2008). Engineering a native homoethanol pathway in *Escherichia coli* B for ethanol production. Biotechnol Lett.

[6] Atsumi S, Wu T-Y, Eckl E-M, Hawkins SD, Buelter T, Liao JC (2010). Engineering the isobutanol biosynthetic pathway in *Escherichia coli* by comparison of three aldehyde reductase/alcohol dehydrogenase genes. Appl Microbiol Biotechnol.

[7] Baez A, Cho K-M, Liao JC (2011). High-flux isobutanol production using engineered *Escherichia coli*: a bioreactor study with in situ product removal. Appl Microbiol Biotechnol.

[8] Nielsen DR, Yoon S-H, Yuan CJ, Prather KLJ (2010). Metabolic engineering of acetoin and meso-2, 3-butanediol biosynthesis in *E. coli*. Biotechnol J.

[9] Xu Y, Chu H, Gao C, Tao F, Zhou Z, Li K (2014). Systematic metabolic engineering of *Escherichia coli* for high-yield production of fuel bio-chemical 2,3-butanediol. Metab Eng.

[10] Hwang HJ, Lee SY, Lee PC. Engineering and application of synthetic nar promoter for fine-tuning the expression of metabolic pathway genes in *Escherichia coli*. Biotechnol Biofuels. 2018;11. https://biotechnologyforbiofuels.biomedcentral.com/articles/10.1186/s13068-018-1104-1. Accessed 17 May 2018. 10.1186/s13068-018-1104-1 PMC5889552 29636821

[11] Nagata S (2001). Growth of *Escherichia coli* ATCC 9637 through the uptake of compatible solutes at high osmolarity. J Biosci Bioeng.

[12] Alterthum F, Ingram LO (1989). Efficient ethanol production from glucose, lactose, and xylose by recombinant *Escherichia coli*. Appl Environ Microbiol.

[13] Lee SY, Chang HN (1993). High cell density cultivation of *Escherichia coli* W using sucrose as a carbon source. Biotechnol Lett.

[14] Arifin Y, Archer C, Lim S, Quek L-E, Sugiarto H, Marcellin E (2014). *Escherichia coli* W shows fast, highly oxidative sucrose metabolism and low acetate formation. Appl Microbiol Biotechnol.

[15] Sabri S, Nielsen LK, Vickers CE (2013). Molecular control of sucrose utilization in *Escherichia coli* W, an efficient sucrose-utilizing strain. Appl Environ Microbiol.

[16] Kazan D, Çamurdan A, Hortaçsu A (1995). The effect of glucose concentration on the growth rate and some intracellular components of a recombinant *E. coli* culture. Process Biochem.

[17] Trinh CT, Unrean P, Srienc F (2008). Minimal *Escherichia coli* Cell for the most efficient production of ethanol from hexoses and pentoses. Appl Environ Microbiol.

[18] Eiteman MA, Lee SA, Altman R, Altman E (2009). A substrate-selective co-fermentation strategy with *Escherichia coli* produces lactate by simultaneously consuming xylose and glucose. Biotechnol Bioeng.

[19] Mazumdar S, Blankschien MD, Clomburg JM, Gonzalez R (2013). Efficient synthesis of l-lactic acid from glycerol by metabolically engineered *Escherichia coli*. Microb Cell Factories.

[20] Lin H, Castro NM, Bennett GN, San K-Y (2006). Acetyl-CoA synthetase overexpression in *Escherichia coli* demonstrates more efficient acetate assimilation and lower acetate accumulation: a potential tool in metabolic engineering. Appl Microbiol Biotechnol.

[21] Luli GW, Strohl WR (1990). Comparison of growth, acetate production, and acetate inhibition of *Escherichia coli* strains in batch and fed-batch fermentations. Appl Environ Microbiol.

[22] Lim HG, Lee JH, Noh MH, Jung GY (2018). Rediscovering acetate metabolism: its potential sources and utilization for biobased transformation into value-added chemicals. J Agric Food Chem.

[23] Mao C, Feng Y, Wang X, Ren G (2015). Review on research achievements of biogas from anaerobic digestion. Renew Sustain Energy Rev.

[24] Schuchmann K, Müller V (2014). Autotrophy at the thermodynamic limit of life: a model for energy conservation in acetogenic bacteria. Nat Rev Microbiol.

[25] Jönsson LJ, Martín C (2016). Pretreatment of lignocellulose: formation of inhibitory by-products and strategies for minimizing their effects. Bioresour Technol.

[26] Li Y, Huang B, Wu H, Li Z, Ye Q, Zhang YHP (2016). Production of succinate from acetate by metabolically engineered *Escherichia coli*. ACS Synth Biol.

[27] Noh MH, Lim HG, Woo SH, Song J, Jung GY (2018). Production of itaconic acid from acetate by engineering acid-tolerant *Escherichia coli* W. Biotechnol Bioeng.

[28] Song HS, Seo HM, Jeon JM, Moon YM, Hong JW, Hong YG, et al. Enhanced isobutanol production from acetate by combinatorial overexpression of acetyl-CoA synthetase and anaplerotic enzymes in engineered *Escherichia coli*. Biotechnol Bioeng. 2018. http://doi.wiley.com/10.1002/bit.26710. Accessed 17 May 2018. 10.1002/bit.26710 29663332

[29] Brown TDK, Jones-Mortimer MC, Kornberg HL (1977). The enzymic interconversion of acetate and acetyl-coenzyme A in *Escherichia coli*. J Gen Microbiol.

[30] Kumari S, Tishel R, Eisenbach M, Wolfe AJ (1995). Cloning, characterization, and functional expression of acs, the gene which encodes acetyl coenzyme A synthetase in *Escherichia coli*. J Bacteriol.

[31] Wolfe AJ (2005). The acetate switch. Microbiol Mol Biol Rev.

[32] Stülke J, Hillen W (1999). Carbon catabolite repression in bacteria. Curr Opin Microbiol.

[33] Enjalbert B, Millard P, Dinclaux M, Portais J-C, Létisse F (2017). Acetate fluxes in *Escherichia coli* are determined by the thermodynamic control of the Pta-AckA pathway. Sci Rep.

[34] Waegeman H, Beauprez J, Moens H, Maertens J, De Mey M, Foulquié-Moreno MR (2011). Effect of iclR and arcA knockouts on biomass formation and metabolic fluxes in *Escherichia coli* K12 and its implications on understanding the metabolism of *Escherichia coli* BL21 (DE3). BMC Microbiol.

[35] Peebo K, Valgepea K, Maser A, Nahku R, Adamberg K, Vilu R (2015). Proteome reallocation in *Escherichia coli* with increasing specific growth rate. Mol BioSyst.

[36] Basan M (2018). Resource allocation and metabolism: the search for governing principles. Curr Opin Microbiol.

[37] Valgepea K, Adamberg K, Nahku R, Lahtvee P-J, Arike L, Vilu R (2010). Systems biology approach reveals that overflow metabolism of acetate in *Escherichia coli* is triggered by carbon catabolite repression of acetyl-CoA synthetase. BMC Syst Biol.

[38] Gimenez R, Felisa Nuñez M, Badia J, Aguilar J, Baldoma L (2003). The gene yjcG, cotranscribed with the gene acs, encodes an acetate permease in *Escherichia coli*. J Bacteriol.

[39] Starai VJ, Celic I, Cole RN, Boeke JD, Escalante-Semerena JC (2002). Sir2-dependent activation of acetyl-CoA synthetase by deacetylation of active lysine. Science.

[40] Starai VJ, Escalante-Semerena JC (2004). Identification of the protein acetyltransferase (Pat) enzyme that acetylates acetyl-CoA synthetase in *Salmonella enterica*. J Mol Biol.

[41] Starai VJ, Gardner JG, Escalante-Semerena JC (2005). Residue Leu-641 of acetyl-CoA synthetase is critical for the acetylation of residue Lys-609 by the protein acetyltransferase enzyme of *Salmonella enterica*. J Biol Chem.

[42] Castaño-Cerezo S, Bernal V, Blanco-Catalá J, Iborra JL, Cánovas M (2011). cAMP-CRP co-ordinates the expression of the protein acetylation pathway with central metabolism in *Escherichia coli*: cAMP-CRP regulates protein acetylation in *E. coli*. Mol Microbiol.

[43] Castaño-Cerezo S, Bernal V, Röhrig T, Termeer S, Cánovas M (2015). Regulation of acetate metabolism in *Escherichia coli* BL21 by protein Nε-lysine acetylation. Appl Microbiol Biotechnol.

[44] Peebo K, Valgepea K, Nahku R, Riis G, Õun M, Adamberg K (2014). Coordinated activation of PTA-ACS and TCA cycles strongly reduces overflow metabolism of acetate in *Escherichia coli*. Appl Microbiol Biotechnol.

[45] Renilla S, Bernal V, Fuhrer T, Castaño-Cerezo S, Pastor JM, Iborra JL (2012). Acetate scavenging activity in *Escherichia coli*: interplay of acetyl-CoA synthetase and the PEP-glyoxylate cycle in chemostat cultures. Appl Microbiol Biotechnol.

[46] Paalme T, Kahru A, Elken R, Vanatalu K, Tiisma K, Raivo V (1995). The computer-controlled continuous culture of *Escherichia coli* with smooth change of dilution rate (A-stat). J Microbiol Methods.

[47] Orth JD, Conrad TM, Na J, Lerman JA, Nam H, Feist AM (2011). A comprehensive genome-scale reconstruction of *Escherichia coli* metabolism—2011. Mol Syst Biol.

[48] Oh M-K, Rohlin L, Kao KC, Liao JC (2002). Global expression profiling of acetate-grown *Escherichia coli*. J Biol Chem.

[49] Salmond CV, Kroll RG, Booth IR (1984). The effect of food preservatives on pH homeostasis in *Escherichia coli*. J Gen Microbiol.

[50] Roe AJ, McLaggan D, Davidson I, O’Byrne C, Booth IR (1998). Perturbation of anion balance during inhibition of growth of *Escherichia coli* by weak acids. J Bacteriol.

[51] Russell JB, Diez-Gonzalez F (1998). The effects of fermentation acids on bacterial growth. Adv Microb Physiol.

[52] Roe AJ, O’Byrne C, McLaggan D, Booth IR (2002). Inhibition of *Escherichia coli* growth by acetic acid: a problem with methionine biosynthesis and homocysteine toxicity. Microbiology.

[53] Engler C, Kandzia R, Marillonnet S (2008). A one pot, one step, precision cloning method with high throughput capability. PLoS ONE.

[54] Sarkari P, Marx H, Blumhoff ML, Mattanovich D, Sauer M, Steiger MG (2017). An efficient tool for metabolic pathway construction and gene integration for *Aspergillus niger*. Bioresour Technol.

[55] Bustin SA (2004). A-Z of quantitative PCR.

