# Supplementary material for: Characterizing the effect of expression of an acetyl-CoA synthetase insensitive to acetylation on co-utilization of glucose and acetate in batch and continuous cultures of E. coli W
Source: Microb Cell Fact. 2018 Jul 9;17:109. doi: 10.1186/s12934-018-0955-2 (PMC6036698; doi:10.1186/s12934-018-0955-2)
Supplement: Supplementary file 1 — Additional file 1: Figure S1. Sequence alignment of Acs of E. coli W and S. enterica LT 2. Residue Lys-609 highlighted by green box represents site of acetylation by Pat, residue Lys-641 highlighted by red box indicates recognition site of Pat for acetylation. [file 12934_2018_955_MOESM1_ESM.docx]

**ADDITIONAL FILE 1**


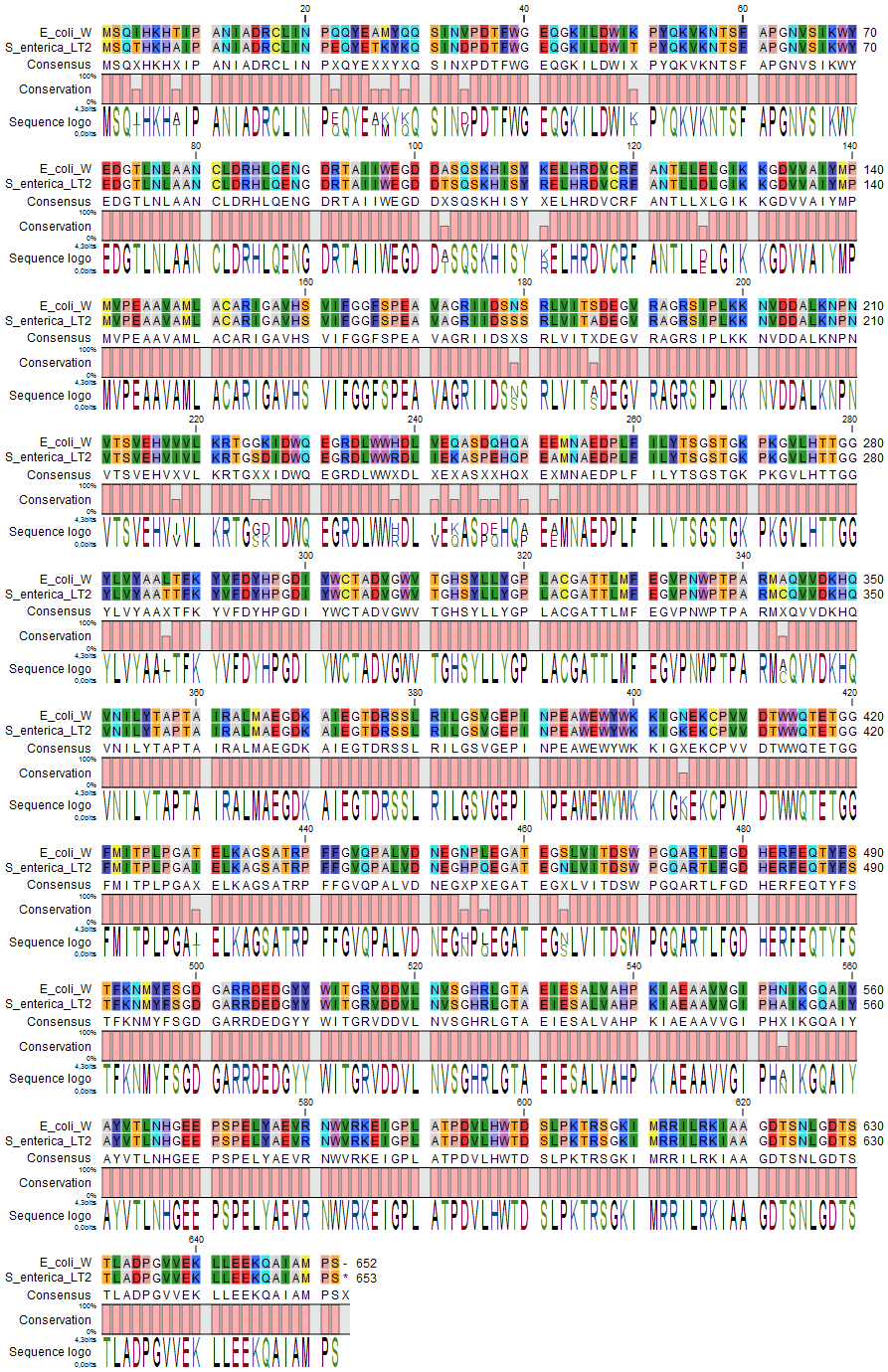


Figure S1: Sequence alignment of Acs of E. coli W and S. enterica LT 2. Residue Lys-609 highlighted by green box represents site of acetylation by Pat, residue Lys-641 highlighted by red box indicates recognition site of Pat for acetylation.
