# Supplementary material for: Characterizing the effect of expression of an acetyl-CoA synthetase insensitive to acetylation on co-utilization of glucose and acetate in batch and continuous cultures of E. coli W
Source: Microb Cell Fact. 2018 Jul 9;17:109. doi: 10.1186/s12934-018-0955-2 (PMC6036698; doi:10.1186/s12934-018-0955-2)
Supplement: Supplementary file 2 — Additional file 2: Table S1. Mean fold change values including standard errors for the four comparisons A, B, C, D (named in the same way as in Figure 1 of the manuscript). Values highlighted in green represent significantly different expression levels (p-value 0.05). Table S2. qPCR program used for gene expression analysis. Lid temperature was set to 95 °C. Table S3. List of primers for gene expression analysis. [file 12934_2018_955_MOESM2_ESM.docx]

**ADDITIONAL FILE 2**

Table S1: Mean fold change values including standard errors for the four comparisons A, B, C, D (named in the same way as in Figure 1 of the manuscript). Values highlighted in green represent significantly different expression levels (p-value 0.05).

| **gene** | **A:**  **ACS_L641P vs. VC, D= 0.2 h^-1^** | | **B:**  **ACS_L641P vs. VC, D= 0.65 h^-1^** | | **C:**  **0.65 vs. 0.2 h^-1^**  **for VC** | | **D:**  **0.65 vs. 0.2 h^-1^**  **for ACS_L641P** | |
| --- | --- | --- | --- | --- | --- | --- | --- | --- |
|  | **mean** | **error** | **mean** | **error** | **mean** | **error** | **mean** | **error** |
| **cra** | 0.69 | 0.13 | 0.57 | 0.06 | 0.47 | 0.07 | 0.40 | 0.06 |
| **acs** | 10.42 | 1.96 | 19.82 | 4.94 | 0.46 | 0.06 | 0.93 | 0.27 |
| **yjcH** | 0.65 | 0.21 | 0.02 | 0.00 | 0.27 | 0.09 | 0.01 | 0.00 |
| **actP** | 0.73 | 0.20 | 0.03 | 0.01 | 0.26 | 0.09 | 0.01 | 0.00 |
| **poxB** | 0.85 | 0.06 | 1.42 | 0.15 | 0.38 | 0.04 | 0.63 | 0.04 |
| **pta** | 1.10 | 0.33 | 0.91 | 0.12 | 0.78 | 0.11 | 0.80 | 0.23 |
| **ackA** | 0.93 | 0.08 | 1.08 | 0.15 | 0.74 | 0.11 | 0.83 | 0.07 |
| **crp** | 0.92 | 0.11 | 0.82 | 0.13 | 0.71 | 0.11 | 0.61 | 0.07 |

Table S2: qPCR program used for gene expression analysis. Lid temperature was set to 95 °C.

45x

| Temperature | Time |
| --- | --- |
| 95 °C | 1:00 min |
| 95 °C | 0:15 min |
| 60 °C | 0:30 min |
| Melt | 0:06 min |

Table S3: List of primers for gene expression analysis.

| **Name** | **Sequence (5’ – 3’)** |
| --- | --- |
| crp_fw | CCATCCAAGAGCACGCTTAT |
| crp_rev | ACTGCCACAGAGCCTTTAAC |
| ackA_fw | GACAACTACGCGACGAAAGA |
| ackA_rev | CACCGATACCACCAGTGAATAC |
| pta_fw | CTACCACGCTAACACCAAAGA |
| pta_rev | AGTTCAGAGACTGGGCAAAC |
| poxB_fw | GCATGTTGATGGGCGATTTC |
| poxB_rev | ATAGCCACCAGCTTTCATCTC |
| actP_fw | TTCTACGCCACCGGATTTATG |
| actP_rev | CCCGCCGCGTCTTTATATT |
| yjcH_fw | GCCACCATCCTGTCGATTATTA |
| yjcH_rev | CGCTGGTGTTCGGATTCA |
| acs_fw | CGCCTACGTCACGCTTAAT |
| acs_rev | AGCGGGCCAATCTCTTTAC |
| cra_fw | GATCTGGAGAACACCAGCTATAC |
| cra_rev | TGATCTTCTGAGCAGGCAATTA |
| tus_fw | GCGTCATTTCCGCCATTTATT |
| tus_rev | TATCGACCTGGTAACACAACAC |
| rrsG_fw | GAGCAAGCGGACCTCATAAA |
| rrsG_rev | GGCATTCTGATCCACGATTACTA |
