# Supplementary material for: Characterizing the effect of expression of an acetyl-CoA synthetase insensitive to acetylation on co-utilization of glucose and acetate in batch and continuous cultures of E. coli W
Source: Microb Cell Fact. 2018 Jul 9;17:109. doi: 10.1186/s12934-018-0955-2 (PMC6036698; doi:10.1186/s12934-018-0955-2)
Supplement: Supplementary file 3 — Additional file 3. Statistical/data evaluation. [file 12934_2018_955_MOESM3_ESM.docx]

**ADDITIONAL FILE 3**

**Statistical/data evaluation**

For off-gas analysis, the proper amount of off-gas had to be determined. First the content of evaporating water in the off-gas (*ex_H2O,out_*) was defined at cultivation set points (37 °C, 2 vvm, 1400 rpm) (Equation 2). *y_O2,in_* is the oxygen content of inlet gas flow and *y_wet_* is the oxygen content of outlet gas flow which were measured as 21.00% and 20.75%, respectively. Secondly for each time point of cultivation the inert gas ratio (*Ra_inert_*) was calculated. In this case inert gases are gases that do not react with the reactor content (all gases except oxygen, carbon dioxide and evaporating water). Therefore, the content of inlet inert gases was divided by the content of outlet inert gases (Equation 3). *y_i_* is the content of component i in percent whereas 1 is indicated as 100% for all gases.

${ex}_{H2O,out}=1-\frac{y_{wet}}{y_{O2, in}}$ (2)

${Ra}_{inert}= \frac{1-\left( \frac{y_{O2,in}+ y_{CO2, in}}{100} \right)}{1-\left( \frac{y_{O2,out}+ y_{CO2,out}}{100} \right)- {ex}_{H2O, out}}$ (3)

With the known in-gas flow rate (*F_in_*) and the determined inert gas ratio, the off-gas flow rate (*F_out_*) can be calculated at each time point.

$$\dot{F}_{out}= \dot{F}_{in}\cdot{Ra}_{in} \left[ \mathrm{sL}h^{-1} \right]$$

(4)

The units for *F_out_* and *F_in_* are standard litre per hour.

For volumetric determination of produced carbon dioxide and consumed oxygen, the net content was converted to decimals, dividing by 100 and multiplied with the off-gas flow rate and time period in hours (Equation 5, 8). This results in the volume of the particular gas indicated as standard litre produced or consumed during a certain time period. The molar amount of this volumes were calculated with the standard volume per mol (Equation 6, 9) to obtain Cmol or Omol. The amount of produced carbon dioxide in Cmol and consumed oxygen in Omol where summed up over the period of cultivation (Equation 7, 10).

$$V_{CO2,t-t-1}=\left( \frac{\left( y_{CO2, out}- y_{CO2,in} \right)\cdot\dot{F}_{out}}{100} \right)\cdot dt [\mathrm{sL}]$$

(5)

$$n_{CO2,t-t-1}=\frac{V_{CO2,t-t-1}}{22.4 L \mathrm{mol}^{-1}} [\mathrm{Cmol}]$$

(6)

$${\sum n}_{CO2, t}={{\sum n}_{CO2, t}-1+ n_{CO2, t}-t-1}_{CO2} [\mathrm{Cmol}]$$

(7)

$$V_{O2,t-t-1}= \frac{\left\{ y_{O2, in}-\left[ y_{O2,out}+\left( y_{O2,dry}-y_{wet} \right) \right] \cdot\dot{F}_{out} \right\}}{100} \cdot dt \left[ \mathrm{sL} \right]$$

(8)

$$n_{O2,t-t-1}=\frac{V_{O2,t-t-1}}{22.4 L \mathrm{mol}^{-1}} [\mathrm{Cmol}]$$

(9)

$${\sum n}_{O2, t}={{\sum n}_{O2, t}-1+ n_{O2, t}-t-1}_{O2} [\mathrm{Cmol}]$$

(10)

The volumetric and specific substrate uptake rates for glucose and acetate were calculated between two sampling points as described below:

$${r_{s}}_{x + 2h}= \frac{(s_{x h}-s_{x + 2h})}{(t_{x+2 h}-t_{x h})}= \frac{ds}{dt} [\mathrm{mmol}L^{-1} h^{-1}]$$

(11)

$$q_{s_{x+2h}}= \frac{{r_{s}}_{x+2h}}{\left( x_{x h}+x_{x+2h} \right)/2} [\mathrm{mmol}g^{-1} h^{-1}]$$

(12)

*s_x h_* are the substrate concentrations in mmol per litre and *x_x h_* the biomass concentrations in gram per litre x hours after inoculation. Since sampling took place at two-hourly intervals, the volumetric and specific uptake rates are indicated as *x + 2 h*.

The specific uptake rates for each individual sampling interval were used to calculate the final weighted specific uptake rates for the entire exponential growth phase (*t_s end_ – t_s1_*):

$$q_{s_{exp}}= \frac{\left( q_{s_{1}}\cdot{dt}_{1} \right)+\left( q_{s_{2}}\cdot{dt}_{2} \right)+... +\left( q_{s_{end}}\cdot{dt}_{s_{end}} \right)}{\left( t_{s_{end}}-t_{1} \right)} \left[ \mathrm{mmol}g^{-1}h^{-1} \right]$$

(13)

*dt* stands for the exact time interval of each respective rate. *t_s end_ – t_1_* is the entire exponential growth phase interval which ranges from 2 or 4 hours after inoculation until depletion of glucose.

The substrate specific yields of biomass, carbon dioxide and oxygen were all calculated related to their amount of mole carbon or mole oxygen. Substrates and biomass were therefore converted from gram per litre (*c_mass_*) into mole per litre (*c_mol_*) und further to mole carbon (*n_mol_*).

For all substrates the total number of carbon atoms (*n*) in the respective substance and the reactor volumes were considered. *M_i_* is the molar mass of component *i*.

$c_{mol}=\frac{c_{mass}}{M_{i}} \left[ mol/l \right]$ (14)

$n_{c}=c_{mol}\cdot n\cdot V_{reactor} \left[ \mathrm{Cmol} \right]$ (15)

The carbon content in dry cell biomass was determined by the previous conducted elementary analysis, as 46.06 % (w/w). *M*_c_ is the molar mass of carbon r the conversion to mole per litre.

$$n_{c}=\frac{c_{mass}\cdot0.4606}{M_{c}}\cdot V_{reactor} \left[ \mathrm{Cmol} \right]$$

(16)

*n*_c_ is the amount of mole carbon in Cmol, that is currently present in the reactor volume *V_reactor_* in litre.

The amount of carbon loss which is caused by sampling was taken into account as well. Therefore, the amount of mole carbon of the respective substance was determined for each sample.

$$n_{c_{sample}}={\frac{n_{c}}{V_{reactor}}\cdot V}_{sample} \left[ \mathrm{Cmol} \right]$$

(17)

The amounts of mole carbon lost during sampling were summed up for each substance and added up with the those present in the fermenter to obtain the actual carbon amount *n_c, actual_*.

$$n_{c_{actual}}=n_{c}+\sum n_{c_{sample}} \left[ \mathrm{Cmol} \right]$$

(18)

All substrate specific yields were calculated comparing the fermentation onset amounts (*0 h*) of substrates, biomass, carbon dioxide, oxygen and NH_3_ with those amounts, at which all substrates were depleted (*End S*).

$$Y_{X/S}= \frac{X_{End S}-X_{0 h}}{S_{0 h}-S_{End S}} \left[ \mathrm{Cmol}\mathrm{Cmol}^{-1} \right]$$

(19)

$$Y_{CO2/S}= \frac{{n_{CO2}}_{End S}- {n_{O2}}_{0 h}}{S_{0 h}-S_{End S}} \left[ \mathrm{Cmol}\mathrm{Cmol}^{-1} \right]$$

(20)

$$Y_{O2/S}= \frac{{n_{O2}}_{End S}- {n_{O2}}_{0 h}}{S_{0 h}-S_{End S}} \cdot2 \left[ \mathrm{Omol}\mathrm{Cmol}^{-1} \right]$$

(21)

$$Y_{NH3/S}= \frac{{m_{NH3}}_{End S}-{m_{NH3}}_{0 h}}{{m_{S}}_{0 h}-{m_{S}}_{End S}} \left[ \mathrm{mmol}g^{-1}h^{-1} \right]$$

(22)

For carbon balancing in the batch processes, the sum of substrates, biomass, metabolites and accumulated carbon dioxide for each sampling point were compared to the sum at fermentation onset (0 h). Total amounts of carbon were used for all components including carbon loss from sampling.

$${C-recovery}_{actual}=\frac{{\sum n}_{c_{actual}}}{\sum n_{c_{0 h}}}\cdot100 \left[ \% \right]$$

(23)

Rates and Yields were calculated in a different way for the chemostat culture. For every sampling point rates were calculated, which were then compared. Equations 2 – 4 were identical, but then equation 24, which does not take *dt* into consideration was used instead of equation 5.

$$V_{CO2,t}=\left( \frac{\left( y_{CO2, out}- y_{CO2,in} \right)\cdot\dot{F}_{out}}{100} \right) [sL h^{-1}]$$

(24)

From this, the molar amount of CO_2_ produced could be calculated in [Cmol/h] with equation 6 and the volumetric CO_2_ production rate [Cmol/l h] was obtained by inclusion of the reactor volume. To avoid wrong results by fluctuations in the offgas signal and as steady state was assumed, the average CO_2_ exhaust from 30 min before and after the sampling in the glucose chemostat was taken into account. For the glucose + acetate A-stats, CO_2_ signals from 5 min before and after sampling were considered.

$$r_{CO2,t}= \frac{n_{CO2,t}}{V_{reactor}}[Cmol L^{-1} h^{-1}]$$

(25)

Volumetric uptake and production rates were obtained by consideration of the dilution rate as well as the molar mass and the number of C atoms in the molecule similar to equation 14 and 15.

$$r_{c, CDM}=\frac{c_{mass}\cdot0.4606}{M_{c}}\cdot D \left[ \mathrm{Cmol}L^{-1} h^{-1} \right]$$

(26)

$$r_{C,i}=\frac{c_{mass}\cdot n_{c,i}}{M_{c,i}}\cdot D \left[ \mathrm{Cmol}L^{-1} h^{-1} \right]$$

(27)

During the conditions when substrate accumulation happened, the uptake concentration was calculated as the difference between the feed concentration and the accumulated substrate. Substrate Yields were further obtained by relation to the substrate uptake *r_Glc_* and/or *r_Ace_*.

$$Y_{C,i/S}=\frac{r_{C,i}}{r_{C, Glc} (+ r_{C,Ace})} \left[ \mathrm{Cmol}\mathrm{Cmol}^{-1} \right]$$

(28)

Summing up all substrate Yields (CO_2_, metabolites, cell dry mass) gives the actual carbon recovery.

$${C-recovery}_{actual}=\sum Y_{C, i/S}\cdot100 \left[ \% \right]$$

(29)

Specific uptake and production rates were obtained by taking the biomass into account.

$$q_{C,i}=\frac{r_{n,i}}{c_{CDM}}\cdot1000 \left[ \mathrm{mmol}g^{-1} h^{-1} \right]$$

(30)

For the glucose chemostat, where all dilution rates were maintained for at least three volume changes, the mean value of three samples taken during steady state was considered. For the glucose + acetate A-stats the mean of two biological replicates were used.
